# Regional Anesthesia in Thyroid Surgery for a Giant Intrathoracic Goiter With Tracheal Compression

**DOI:** 10.7759/cureus.76495

**Published:** 2024-12-27

**Authors:** Dragan Milosevic, Suzana Sobot Novakovic, Anita Djurdjevic Svraka, Dragan Švraka

**Affiliations:** 1 Anesthesiology and Critical Care, University Clinical Center of the Republic of Srpska, Banja Luka, BIH; 2 Surgery, Faculty of Medicine, University of Banja Luka, Banja Luka, BIH; 3 Center for Biomedical Research, Faculty of Medicine, University of Banja Luka, Banja Luka, BIH; 4 Anesthesiology, Resuscitation, and Intensive Care, Gradiška General Hospital, Gradiska, BIH; 5 Faculty of Medicine, University of Banja Luka, Banja Luka, BIH; 6 Anesthesiology and Critical Care, Faculty of Medicine, University of Banja Luka, Banja Luka, BIH

**Keywords:** intrathoracic goiter, regional anesthesia, severe tracheal compression, superficial cervical plexus block, thyroid surgery

## Abstract

Cervical plexus block (CPB), like other types of regional anesthesia, represents an alternative anesthetic technique in those cases where the performance of general anesthesia (GA) carries an increased risk both for the patient and the outcome of the operative treatment. It has traditionally been used for years in carotid surgery as an alternative to GA, especially due to the possibility of superior monitoring - the awake patient. However, its effectiveness has been proven in other types of neck surgery, primarily in thyroid surgery, neck dissections, tracheostomy, central venous catheter insertion, clavicle surgery, etc. In most cases, it provides adequate and satisfactory analgesia of the anterolateral side of the neck (C1-C4 roots), at the same time avoiding all the negative effects on the patient that GA entails. Superficial block of the cervical plexus (SBCP) is a variant known for its simplicity and low number of complications. It can be performed traditionally with the help of external landmarks or with ultrasound-guided orientation.

The case report describes the anesthesiology challenges of maintaining the airway caused by a high degree of compression by the retrosternal goiter and the possibility of performing such an operation under regional anesthesia. In this case, a male patient in his seventies presented with a giant retrosternal goiter and symptoms associated with tracheal compression. Due to significant tracheal narrowing, induction of general endotracheal anesthesia was not possible. The surgical treatment was performed through a low-collar incision with neck extension under bilateral superficial cervical plexus (BSCPB) block anesthesia. The study concludes that BSCPB can provide a satisfactory degree of analgesia for operative procedures like this, where GA poses a high risk.

## Introduction

Intrathoracic goiter is a phenomenon when individual lobes of the thyroid gland or the entire thyroid gland cross the border of the visceral space of the neck, enter the space of the anterior mediastinum, and occupy a position behind the sternum. Some authors believe that a retrosternal goiter is any goiter that propagates below the level of the upper aperture of the thorax. In contrast, some believe that for a goiter to be classified as retrosternal or intrathoracic, at least half of the mass must be located within the thorax [1]. Parts of the goiter can be positioned along the trachea descending to the bifurcation of the trachea, as well as along the trachea. In some cases, it can be in contact with the arch of the aorta and the surrounding vascular elements. Compression of the trachea is most often caused in the neck and upper thoracic aperture and less often in the mediastinum by a large nodular goiter [2]. The long-term pressure on the trachea causes deformation, degeneration, and softening of the cartilage, which is known as tracheomalacia. Since the thyroid gland is one of the organs with the highest blood supply, and vascularization increases with the growth of the goiter itself, the control of bleeding in such intrathoracic goiter operations is a significant challenge [1-2]. This case represents a big challenge due to the increased risk of induction into general endotracheal anesthesia. For this reason, an operation under regional anesthesia with the use of analgesia was considered and performed.

Different techniques of regional anesthesia have demonstrated a reduction in pain and an opioid-sparing effect during and after surgery. Very often, regional anesthesia represents a good alternative in cases where the performance of general anesthesia (GA) is associated with a high risk for the patient and the final outcome of the treatment.

The cervical plexus block (CPB) provides effective anesthesia and analgesia for the head and neck region, with its most common clinical application being carotid endarterectomy (CEA) [3]. According to a systematic review and meta-analysis by Wilson et al., bilateral superficial cervical plexus block (BSCPB) is a commonly used regional anesthesia technique for providing analgesia in thyroid surgery. The same author concludes that BSCPB is a procedure with a low rate of serious complications and that the risks of phrenic nerve palsy and total spinal anesthesia from injection into a *dural cuff* are restricted to blockade of the deep cervical plexus only; diffusion of the local anesthetic is not sufficient to account for these phenomena with the superficial technique [4].

## Case presentation

A 72-year-old male patient with a giant goiter was admitted to our hospital, presenting with signs and symptoms consistent with severe tracheal obstruction. The patient’s medical history included chronic obstructive pulmonary disease (COPD), arterial hypertension, and cholelithiasis. The patient’s body mass index (BMI) was 28.2, with a body weight of 97 kg and an American Society of Anesthesiologists (ASA) score of III.

Imaging and diagnosis

**Figure 1:**
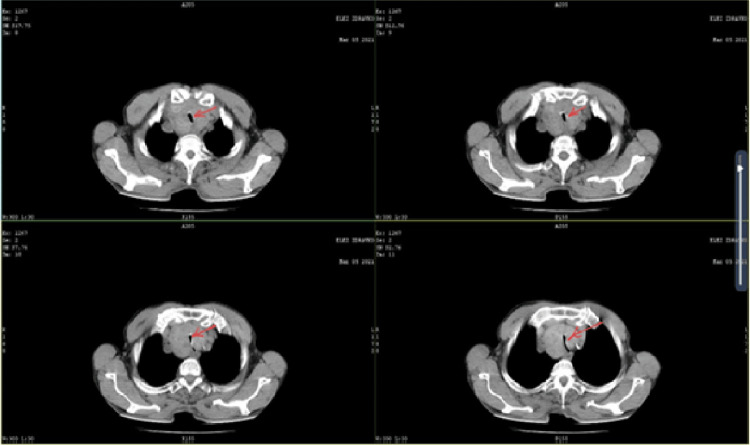
Computed tomography (CT) of the neck and chest, taken four days before surgical treatment, showing tracheal compression and airway obstruction caused by the surrounding thyroid gland tissue.

Computed tomography (CT) imaging revealed enlarged thyroid lobes, with the right lobe having the largest diameter of 13.5 cm. The thyroid gland extended intrathoracically, with dimensions of 8.5 cm x 5 cm x 4 cm. Evidence of tracheal narrowing was observed, with the narrowest segment measuring 2.5 mm, located 2 cm proximal to the tracheal bifurcation (Figure 1). A previous biopsy confirmed the presence of a benign nodule.

Anesthetic management

**Figure 2:**
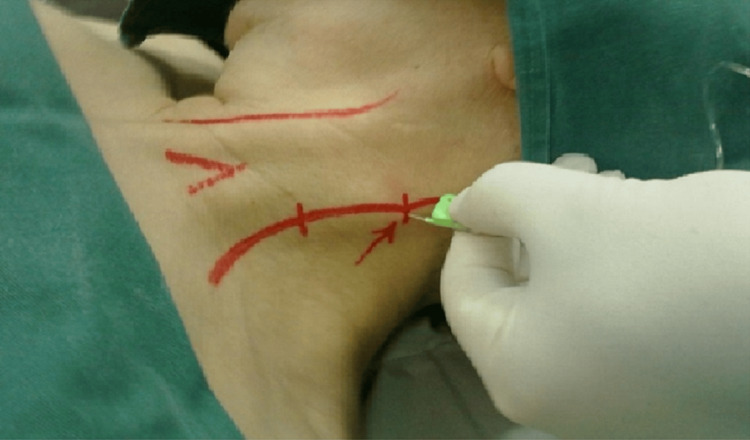
Landmark for performing the superficial cervical plexus block: infiltration of the posterior border of the sternocleidomastoid muscle using the redirection technique.

Due to significant tracheal compression, general endotracheal anesthesia was deemed unfeasible. Therefore, surgical intervention was performed under BSCPB anesthesia. BSCPB was executed by infiltrating 15 mL of 1.7% lidocaine on each side of the posterior edge of the sternocleidomastoid muscle (Figure 2). Additionally, local infiltration with 2 mL of 1.7% lidocaine was administered at the incision site.

Intraoperative monitoring and sedation

Standard intraoperative monitoring included non-invasive blood pressure (NIBP), oxygen saturation, and electrocardiogram (ECG) lead II. The patient maintained spontaneous ventilation with oxygen supplementation at a rate of 4 L/minute. Minimal sedation and analgesia were provided with 2 mg of midazolam and 75 mcg of fentanyl.

Surgical procedure

**Figure 3:**
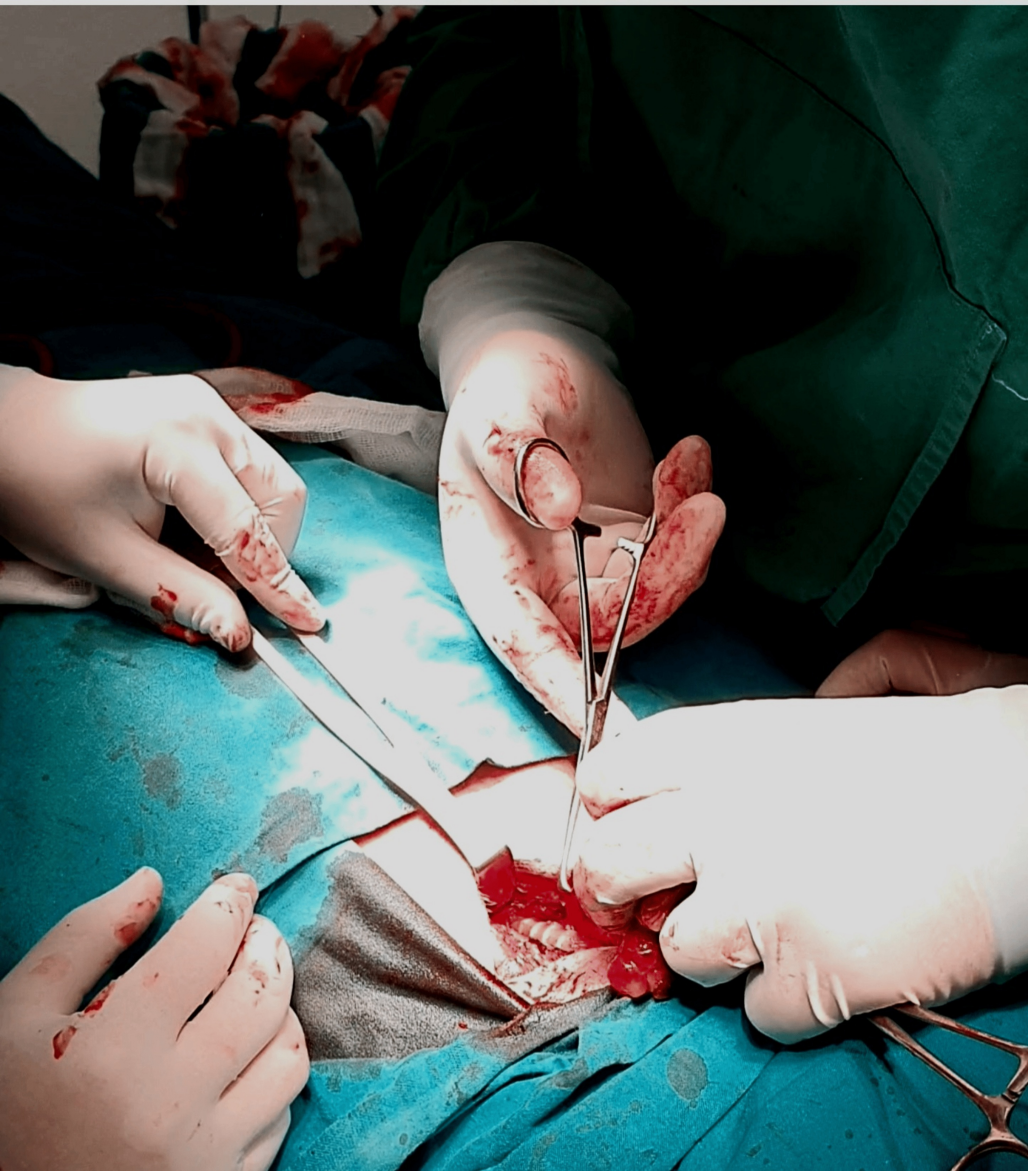
Intraoperative dissection of the trachea (the ring structure is visible).

A low-collar incision was made, with the neck in an extended position (Figure 3). The surgical procedure used standard techniques for goiter resection, including the LigaSure Small Jaw Sealer/Divider (LF1212A). The procedure lasted 100 minutes, during which hemodynamic and respiratory stability were maintained, with oxygen saturation at 96%. The patient remained conscious and reported minimal pain, with an average Visual Analog Scale (VAS) score of 2.5.

Outcome and postoperative course

**Figure 4:**
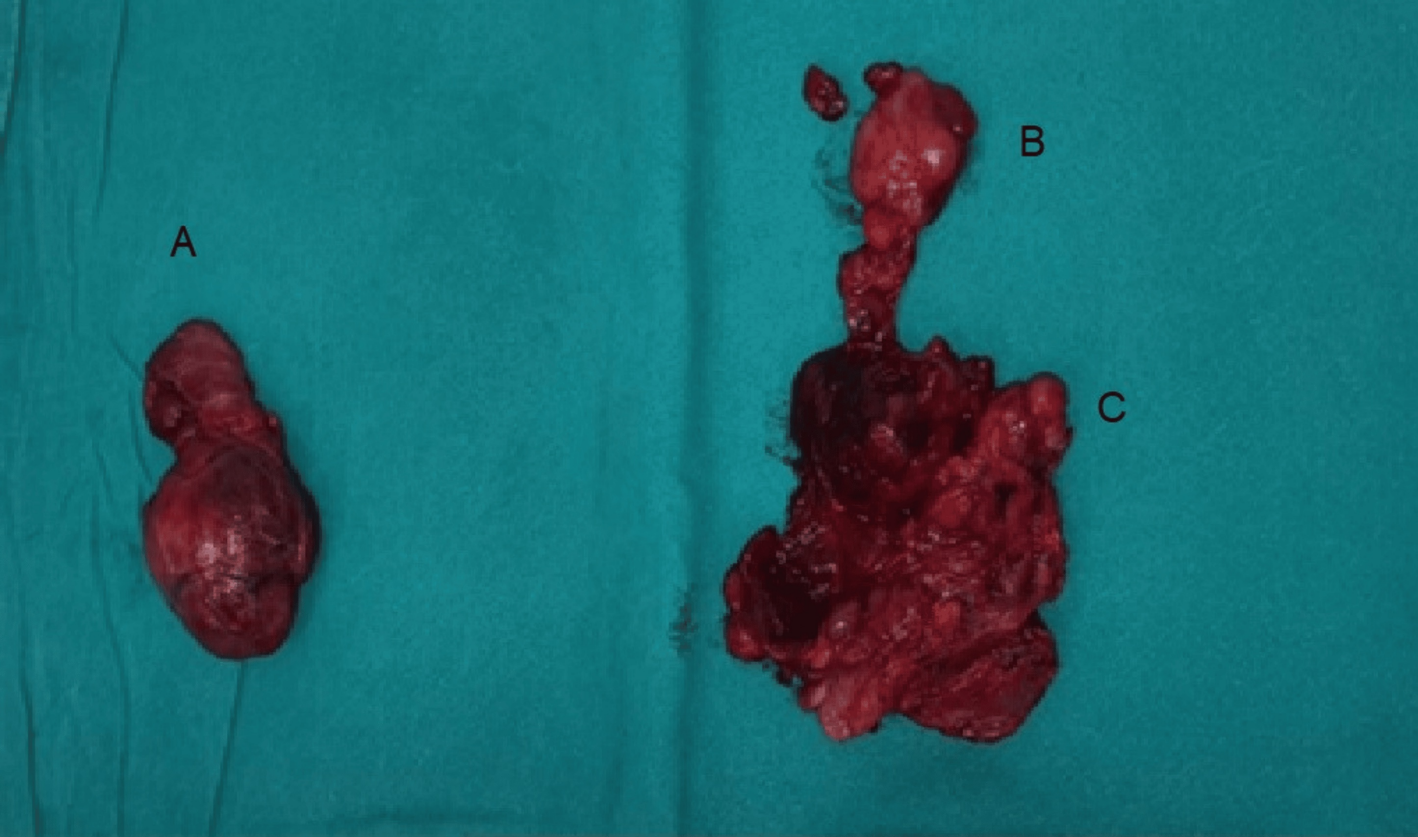
Left lobe (A) measures 75 mm x 40 mm and 50 mm x 30 mm, the right lobe (B) measures 50 mm x 30 mm x 15 mm, and the intrathoracic segment (C) measures 85 mm x 50 mm x 40 mm (information provided by the pathologist).

The surgical intervention resulted in complete removal of the giant thyroid gland (Figure 4). The airway was successfully preserved, obviating the need for an emergency tracheotomy. Intraoperative assessment revealed no tracheomalacia or tracheal stenosis, with compression being the primary cause of airway compromise. Both recurrent laryngeal nerves were preserved, and phonation remained intact. All four parathyroid glands were identified and preserved.

Postoperative laboratory evaluations demonstrated the following serum calcium levels: 1.86, 1.77, and 1.71 mmol/L. On the ninth postoperative day, calcium levels improved to 2.00 mmol/L (ionized calcium: 1.26 mmol/L), parathyroid hormone (PTH) was 12.5 pg/mL, free thyroxine (FT4) was 0.63 ng/dL, and thyroid-stimulating hormone (TSH) was 2.24 μIU/mL. No clinical signs of hypocalcemia were observed, confirming preserved parathyroid gland function. Postoperatively, in the first 24 hours, satisfactory analgesia was achieved with a combination of paracetamol and ketonal, without the use of opiates, with an average VAS score of 4.

## Discussion

SCPBs are widely employed in head and neck surgeries to ensure adequate anesthesia and analgesia. Despite the anatomical complexity of the neck region, which contains multiple sensitive structures and fascial layers, SCPB remains a reliable technique. Traditionally used for awake carotid endarterectomies, SCPB has found applications in various surgical specialties, either as an adjunct to GA as well as sole technique [3].

Cervical block, superficial or deep, unilateral or bilateral, as an anesthetic technique in thyroid surgery, deserves attention. This block is not used as a routine method, but as an alternative when GA poses too great a risk. The decision on its application should be made based on many factors such as the patient's general condition, the existence of a risk of difficult intubation, as well as the anesthesiologist's familiarity with this type of regional anesthesia. 

There are no generally accepted criteria for defining retrosternal goiter (also known as intrathoracic or mediastinal goiter). While some authors believe that retrosternal goiters should include all those in which the lower poles propagate below the level of the upper aperture of the thorax, others point out that more than half of the mass of the goiter must be located in the thorax. This is why the frequency of such goiters is estimated differently, ranging from 1% to 45% [1,2].

CPB can represent an alternative anesthetic technique to GA for thyroid procedures in situations where, due to the size of the goiter that compresses the trachea, it is impossible to perform safe intubation to administer general endotracheal anesthesia and avoid a tracheotomy for intubation. Its analgesic efficacy is widely documented in the available literature, but there are no papers showing its utility as a solo technique in thyroid surgery.

Wilson et al. in meta-analysis and systematic review concluded that BSCPB offers superior postoperative analgesia with a reduction in opioid use, reduction in postoperative nausea and vomiting (PONV), and improvement in VAS scores, but as an adjunct to GA [4].

Sun et al. used it in combination with GA in a case report from 2021, involving a patient with acromegaly and a giant goiter, and emphasized the importance of a carefully designed airway management strategy and close communication among the multidisciplinary surgical team [5].

Mayhew et al., in their meta-analysis and systematic review, found that BSCPB offers analgesic efficacy in the early postoperative period for up to 24 hours after thyroid surgery, with a reduced length of hospital stay, but without any beneficial effect on PONV [6].

An SBCP is successfully performed by subcutaneous infiltration of a local anesthetic along the posterior border of the sternocleidomastoid muscle in its middle part. An ultrasound-guided technique can also be used, which is more appropriate for deep or intermediate variants of the block. Suh et al. found that SPCB is a more effective technique than combined superficial and deep CPB in reducing pain during and immediately after thyroidectomy [7].

Pandit et al., in a prospective randomized study, found no differences in analgesic efficacy between a superficial and combined block in carotid surgery and preferred the superficial variant due to the lower incidence of complications [8]. Similarly, Messner et al., in a prospective randomized controlled trial, favored SCPB for postoperative pain control after CEA, with a clinically significant opioid-sparing effect [9].

Contrary to the stated views, some authors do not recommend the routine use of SBCP for pain control in thyroid surgery, as they found the effect on pain reduction to be too small to be of clinical relevance. They recommend further trials to evaluate the dose-response relationship and incidence of PONV with this regimen, as suggested by Warschkow et al. in their meta-analysis of randomized controlled trials [10].

In conditions where tracheal compression is present and intubation for general endotracheal anesthesia is impossible, it is justified to free the trachea first under cervical block anesthesia. This approach ensures that if there are complications with the cervical anesthesia or the patient’s breathing, a tracheotomy can be quickly and safely performed, along with jet ventilation. Regional cervical block anesthesia is justified in such situations to avoid additional injury to the trachea by performing a tracheotomy without knowing how the tracheal tissue will react postoperatively after decompression and what the healing process will be like.

## Conclusions

Based on the findings above, we can conclude that operative treatment of giant retrosternal intrathoracic goiter can be performed without tracheotomy under conditions of BSCPB. A high-quality diagnosis is needed, starting with a contrast-enhanced computed tomography scan to determine the size, position, and relationship of the goiter with other structures. Cooperation with the patient is essential, as the patient remains awake and alert throughout the entire procedure. Tracheotomy should always be considered a backup option if complications with breathing or pain arise perioperatively. The most important factor is good cooperation and communication between the surgeon, anesthesiologist, and patient during the procedure.

## References

[1] Knobel M (2021). An overview of retrosternal goiter. J Endocrinol Invest.

[2] Doulaptsi M, Karatzanis A, Prokopakis E, Velegrakis S, Loutsidi A, Trachalaki A, Velegrakis G (2019). Substernal goiter: treatment and challenges. Twenty-two years of experience in diagnosis and management of substernal goiters. Auris Nasus Larynx.

[3] Kim JS, Ko JS, Bang S, Kim H, Lee SY (2018). Cervical plexus block. Korean J Anesthesiol.

[4] Wilson L, Malhotra R, Mayhew D, Banerjee A (2023). The analgesic effects of bilateral superficial cervical plexus block in thyroid surgery: a systematic review and meta-analysis. Indian J Anaesth.

[5] Sun X, Chen C, Zhou R, Chen G, Jiang C, Zhu T (2021). Anesthesia and airway management in a patient with acromegaly and tracheal compression caused by a giant retrosternal goiter: a case report. J Int Med Res.

[6] Mayhew D, Sahgal N, Khirwadkar R, Hunter JM, Banerjee A (2018). Analgesic efficacy of bilateral superficial cervical plexus block for thyroid surgery: meta-analysis and systematic review. Br J Anaesth.

[7] Suh YJ, Kim YS, In JH, Joo JD, Jeon YS, Kim HK (2009). Comparison of analgesic efficacy between bilateral superficial and combined (superficial and deep) cervical plexus block administered before thyroid surgery. Eur J Anaesthesiol.

[8] Pandit JJ, Bree S, Dillon P, Elcock D, McLaren ID, Crider B (2000). A comparison of superficial versus combined (superficial and deep) cervical plexus block for carotid endarterectomy: a prospective, randomized study. Anesth Analg.

[9] Messner M, Albrecht S, Lang W, Sittl R, Dinkel M (2007). The superficial cervical plexus block for postoperative pain therapy in carotid artery surgery. A prospective randomised controlled trial. Eur J Vasc Endovasc Surg.

[10] Warschkow R, Tarantino I, Jensen K, Beutner U, Clerici T, Schmied BM, Steffen T (2012). Bilateral superficial cervical plexus block in combination with general anesthesia has a low efficacy in thyroid surgery: a meta-analysis of randomized controlled trials. Thyroid.

